# Demonstration of Hepatic Vein Abnormalities Using Contrast-Enhanced Sonography in Liver Diseases

**DOI:** 10.3390/diagnostics15060709

**Published:** 2025-03-12

**Authors:** Hiroko Naganuma, Hideaki Ishida

**Affiliations:** 1 Department of Gastroenterology, Yokote Municipal Hospital, Yokote 013-8602, Japan; 2 Department of Gastroenterology, Akita Red Cross Hospital, Akita 010-1495, Japan

**Keywords:** contrast-enhanced sonography (CEUS), liver, liver tumor, vascular anomalies, hepatic vein, transit time

## Abstract

Contrast-enhanced US (CEUS) is now widely used to observe the hemodynamics of the liver. The CEUS diagnosis mainly consists of evaluating hepatic artery and portal vein flow changes in liver diseases, but it has not been widely used for the diagnosis of hepatic venous (HV) abnormalities in the clinical setting. For this background, this review tried to reconsider this problem. In short, observing HV CEUS findings, especially HV transit time, serves to largely narrow the differential diagnosis and increase the diagnostic confidence of the CEUS. However, diagnosing HV CEUS diagnosis in a wide range of liver diseases requires understanding of vascular anatomy of the upper abdomen and vascular structure of each disease. Additionally, interpreting CEUS findings of HCC should be prudent, because its drainage vessels change according to the histological progression, from the HV to the portal vein. Thus, the most important way of making use of the CEUS information is interpreting it in conjunction with the clinical data.

## 1. Introduction

The gold standard for the diagnosis of hepatic venous abnormalities is balloon occlusion venography [1]. This concept remains unchanged until now. This method, which has remained unchanged until now, can pick up many minute changes (stenosis, dilatation, or thrombosis) of the hepatic veins and can directly measure hepatic venous pressure, which cannot be obtained using any other method. However, this method is now rarely performed for diagnostic purposes because it is invasive and painful for the patient. It requires the use of large amounts of contrast media and involves radiation exposure during the examination [1]. So-called noninvasive imaging methods (ultrasound (US) [2,3], X-ray CT [4,5], MRI [6], and positron-emission CT [7]) that have emerged more recently have their own advantages, and Doppler US seems to be the most widely used method for evaluating hepatic venous hemodynamics in general clinical practice [8]. In addition to observing changes in the diameter of hepatic veins in real time, this method enables us to grasp changes in venous flow according to cardiac function through the use of the FFT waveform [9,10]. One of the major drawbacks of this method is the insufficient spatial resolution of the displayed colored information; consequently, it is unsuitable for detailed diagnosis, such as confirmation of the presence or absence of minute vascular shunts [8,11]. Contrast-enhanced US (CEUS), which has become widely used in recent years, is expected to greatly reduce the above disadvantages of Doppler US [12,13]. However, CEUS has not been widely used for the diagnosis of hepatic venous abnormalities in the clinical setting [14]. For this reason, we endeavored to reconsider this problem in this review.

## 2. CEUS of Hepatic Veins

**Figure 1:**
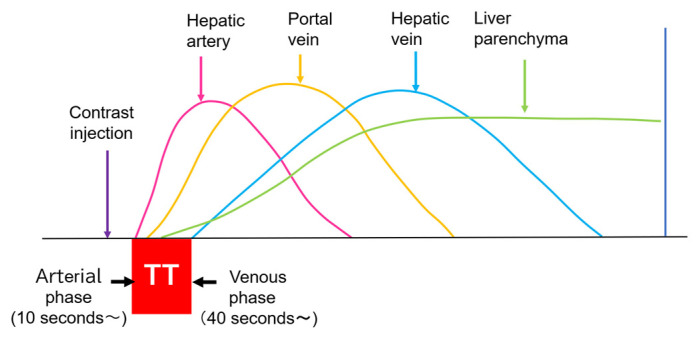
CEUS of the intrahepatic vascular system: Hepatic artery begins to be enhanced at about 10 s after contrast injection. Portal vein begins to be enhanced at about 30 s after contrast injection. Hepatic vein begins to be enhanced at about 40 s after contrast injection. TT, transit time.

**Figure 2:**
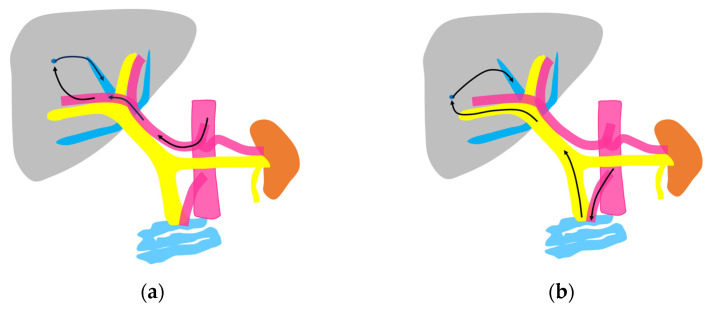
Schematic drawing of two routes of the contrast medium to the hepatic vein: (**a**) The hepatic artery route in which the contrast medium passes through the celiac artery, HA, the peripheral small intrahepatic arterial branches, and the sinusoids; finally, it arrives at the central HVs (arrows). (**b**) The portal vein route in which the contrast medium passes through the superior mesenteric artery, bowel wall, and superior mesenteric vein and then travels to the main portal vein, intrahepatic portal branches, sinusoids, and the central HVs (arrows).

Currently used microbubbles do not penetrate into the hepatic parenchyma, although they cover the entire liver and appear to display the whole hepatic parenchyma [14]. Strictly speaking, the postvascular phase of a CEUS image is merely a collection of microbubbles passing through peripheral microvessels. However, unlike CT and MRI, in which a contrast agent passes into the liver parenchyma (interstitium), this characteristic of CEUS reflects the purely intravascular movement of the contrast medium and thus provides accurate and detailed vascular information on the liver [14]. In CEUS diagnosis, we must keep in mind the concepts of CEUS enhancement patterns [15,16] and hepatic vein (HV) transit time [17,18,19]. As mentioned above, the contrast medium passes through the vessels, and this dynamic imaging permits us to observe hemodynamic changes in the liver. Based on these findings, the American College of Radiology (ACR) released the CEUS Liver Imaging Reporting and Data System (LI-RADS) in 2016 for the first time [15,16]. It states that CEUS images change according to the vascular phase: this classification was initially used for liver tumor diagnosis, but this concept can be applied to all hepatic vascular abnormalities. In the CEUS diagnosis, the arterial phase begins at approximately 10 s, and the portal phase begins at approximately 30 s after the contrast injection [17,19,20]. The HV transit time is the sum of the time taken for the microbubble to travel from the hepatic artery (HA) to the HV, after passing through the peripheral vessels [20]. If we compare the HA onset time with the HV onset time, we see that the HV onset time is approximately 30 s later than the HA onset time (Figure 1). To explain this time delay, the vascular anatomy of the upper abdomen is indispensable. There are two contrast passage routes. One is via the celiac artery–HA route. The contrast medium then passes to the peripheral small intrahepatic arterial branches and through the sinusoids; finally, it arrives at the central HVs. The other route is via the superior mesenteric artery-bowel wall–superior mesenteric vein route. The contrast medium then passes to the main portal vein, the intrahepatic portal branches, and the sinusoids, and the central HVs (Figure 2a,b). The time delay from the beginning of HA enhancement to the enhancement onset of the major HVs (right, middle, and left) is the transit time [19,20]. The enhancement onset time of the three HVs is reported to be the same [19]. After which, the HV enhancement continues because of the mixture of the contrast medium of the two routes described above. The transit time changes partially or completely when pathologic changes occur during this process. It has been reported that liver cirrhosis or liver tumors accelerate transit time. However, this idea of altered transit time should have broader applicability, and this is discussed in the following sections.

## 3. HV Hemodynamic Changes in Liver Tumors

### 3.1. Hepatocellular Carcinoma (HCC)

**Figure 3:**
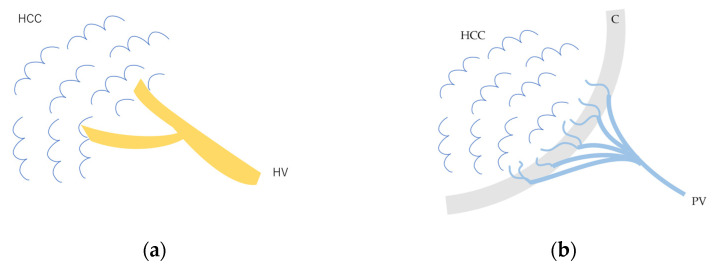
Schematic drawing of afferent and efferent vessels of HCC: (**a**) In early HCC and some well-differentiated HCC, the afferent vessel is HA. HVs remain within the lesion, and blood flow drains from these veins into the HVs via sinusoids. (**b**) In advanced moderately to poorly differentiated HCC, the afferent vessel is HA, and blood flow drains into the portal vein via capillarized sinusoids. HCC, hepatocellular carcinoma; HV, hepatic vein; C, capsule; PV, portal vein.

**Figure 4:**
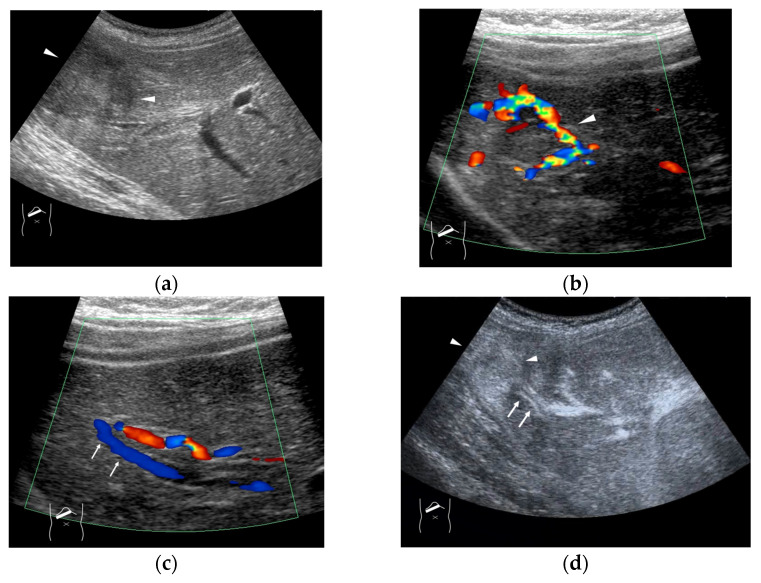
Representative case of surgically resected well-differentiated HCC arising from minimally fibrotic (F1) liver: (**a**) B-mode reveals HCC in segment 6 (arrow heads). (**b**,**c**) Color Doppler shows tumor vascularities in a basket pattern (arrow heads) and a hepatic vein (arrows). (**d**) CEUS reveals a markedly enhanced HCC lesion (arrow heads). A hepatic vein (arrows) is enhanced immediately after tumor enhancement.

The drainage vessels of HCC are quite complex [21,22,23,24,25]. To understand this, it is essential to understand the evolution of the afferent and drainage vessels of the hepatocarcinogentic nodule, from the dysplastic nodule (DN) (pre-HCC stage), to early HCC (eHCC), and to advanced HCC (moderately to poorly differentiated). To briefly summarize, in the low-grade DN, portal blood flow is slightly reduced compared to the surrounding parenchyma, and blood flow in the HA is similar to that in the surrounding liver tissue. In the high-grade DN, portal blood flow and blood flow in the HA are both reduced compared to the surrounding tissue, and only a few neovascularized arteries appear [21,22,23,24,25]. In eHCC, this appearance of high-grade DN hemodynamics is essentially preserved but becomes more pronounced. In advanced HCC, the afferent HA and portal vein disappear, leaving only neovascularized arteries [21,25]. In advanced HCC, however, the HV within the lesion disappears, and blood flow drains into the portal vein via capillarized sinusoids (Figure 3a,b). Generally speaking, these studies have been focused on cirrhosis-related multistep hepatocarcinogentic nodules. However, in eHCC and some well-differentiated HCC, the HVs remain within the lesion, and blood flow drains from these veins into the HVs via sinusoids [26,27] (Figure 4a–d). Again, we must emphasize that the classic theory that the portal vein is the drainage vessel of malignant tumors and the HV is the drainage vessel of benign tumors is incorrect [28,29]. Future studies are needed to determine whether this unusual pattern occurs in different environments of HCC, such as those arising from minimally fibrotic [30,31] or normal livers [32,33]. A simple way to prevent misdiagnosis is to judge the draining mode in conjunction with interpretation of the CEUS findings of the lesion on the basis of CEUS LI-RADS*, and if the diagnosis is difficult, short-term follow-up is recommended. For a detailed diagnosis of the afferent and drainage vessels of liver tumors, CTHA and CTPV (X-ray CT combined with angiography) are the best methods, although they are invasive techniques.

*CEUS LI-RADS: This is classified based on the probability of a lesion being HCC based on arterial phase hyper-enhancement, wash-out, and other additional features, and the classification ranges between LR-1 (absolutely benign) and LR-5 (absolutely HCC). The higher the classification, the higher the probability of the lesion being HCC [34,35].

### 3.2. Liver Metastasis

**Figure 5:**
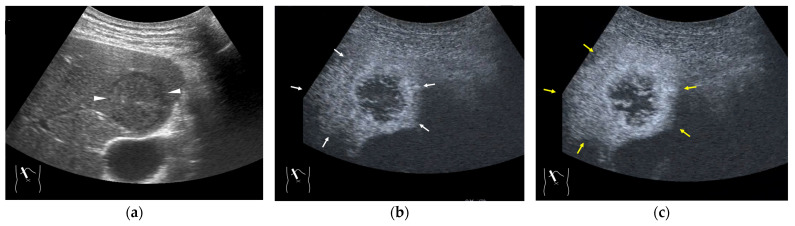
Representative case of surgically resected solitary metastasis from small bowel sarcomatous adenocarcinoma: (**a**) The lesion (arrow heads) is well demarcated on B-mode US. (**b**,**c**) It is homogeneously hyper-enhanced (white arrows) in the arterial phase, and the tumor size appears to increase (yellow arrows), probably due to many surrounding fine vascular shunts.

As mentioned above, the draining vessels of HCC also change significantly according to the histological changes in the background hepatic parenchyma. Thus, the CEUS findings must be comprehensively judged in combination with other clinical data. This complexity of the draining vessels also occurs in other malignancies, such as liver metastasis. Liver metastases [36] basically arise from an almost normal liver parenchyma without fibrosis: thus, there is no compression of the vasculature by fibrosis, as is seen in liver cirrhosis. The draining vessels of liver metastases are basically HVs, but due to the compression of the surrounding tissues by the tumor, numerous shunts are formed, some of the draining vessels may be portal veins, which complicates the process [37] (Figure 5a–c). According to some recent studies, hepatic metastases are divided into three types according to their growth patterns (desmoplastic, pushing and replacement patterns) [36,38]. The question of whether there is an association between the liver metastasis growth pattern and the draining vessel pattern is an important one that needs to be answered in the near future.

### 3.3. Benign Liver Tumors

**Figure 6:**
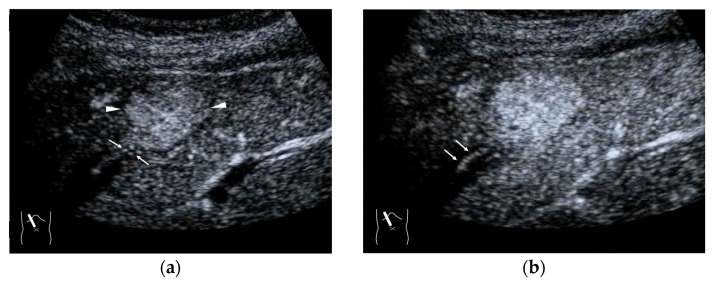
Representative case of FNH: (**a**,**b**) CEUS shows a spoke-wheel appearance in the arterial phase (arrow heads). It also shows early draining into the hepatic vein (arrows).

**Figure 7:**
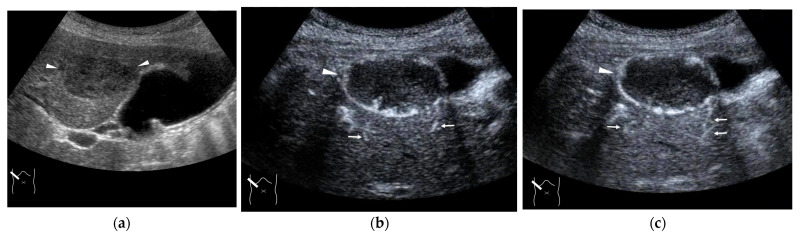
Representative case of a hepatic hemangioma with draining vessels: (**a**) B-mode shows a hypoechoic mass in segment 5 (arrow heads). (**b**,**c**) CEUS reveals the mass to be a hemangioma with a fill-in appearance (arrow heads) and venous drainage (arrows).

In contrast to HCC, the formation of draining vessels has been relatively well clarified in benign tumors [39]. Therefore, in the CEUS image, there is clear evidence of contrast outflow into the HVs immediately or slightly after the lesion is enhanced in the arterial phase. An early outflow of the contrast medium into a large hepatic vein is an important finding in AML [40] and FNH [41,42] (Figure 6a,b) and is useful in the differential diagnosis. However, in most hemangiomas, the outflow is slow and indistinct. Because the draining vessels of hepatic hemangiomas generally do not have such large draining vessels and blood flow seeping out of the lesion flow into the nearby HVs [43], the efflux of the contrast medium in the CEUS image is relatively slow, and the nearby HVs become enhanced sometime after the lesion has been enhanced in the CEUS image (Figure 7a–c). Notable, however, with regard to hepatic hemangiomas, is the presence of a hypervascular hemangioma called a flash hemangioma (or high-flow hemangioma) in a small number of cases: it is imaged as a markedly enhanced lesion with a rapid outflow of blood due to arterio-portal (A-P) or arterio-venous (A-V) shunts at the tumor margins (Figure 7a). In summary, the majority of hemangiomas show rim enhancement, show a fill-in appearance, and are classified as LR-1 by CEUS LI-RADS [44]. The HV is slowly enhanced, while the hypervascular hemangiomas are strongly enhanced in the arterial phase immediately, and the draining vessel is the portal vein or HV through fine A-P or A-V shunts; thus, the diagnosis of hemangiomas in this group requires a summarized analysis of many tests [44].

## 4. HV Hemodynamic Changes (Vascular Shunts) in Non-Tumor-Related Livers

The diagnosis of vascular shunts in a tumor-free liver requires a completely different approach from that for tumor-related vascular shunts [23]. The non-tumor-related vascular shunt group can be roughly divided into two subgroups: (1) those in which a wide spectrum of vascular shunts (A-Ps, A-Vs, P-Vs, or V-Vs) coexist and (2) those in which only one type of vascular shunt is present. The former group is almost exclusively seen in patients with hereditary hemorrhagic telangiectasia (HHT) or similar diseases, while the latter group is extremely diverse, with different etiologies and clinical manifestations depending on the shunts [45].

### 4.1. HHT-Related HV Abnormalities

**Figure 8:**
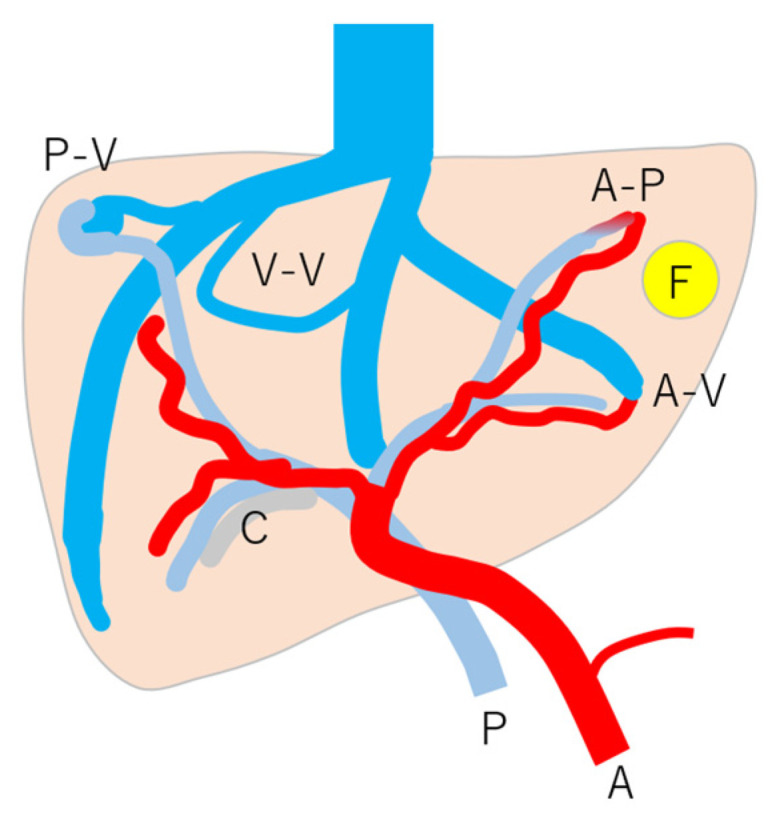
Schematic drawing of vascular changes in HHT: note that HHT is affected by many kinds of vascular changes, but the mode and degree of change are according to individuals and their age. A, c artery; P, portal vein; C, cholangitis; F, focal nodular hyperplasia; A-V, arterio-venous shunts; A-P, arterio-portal shunt; P-V, portal-venous shunt; V-V, veno-venous shunt. (Cited from [45] with adequate modification.)

**Figure 9:**
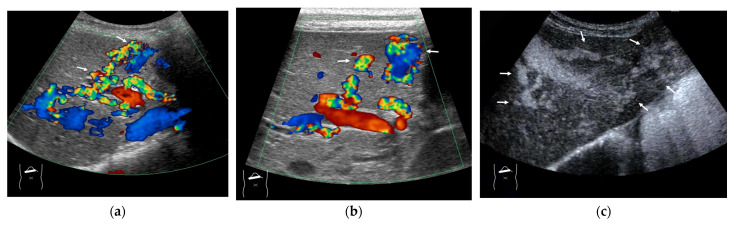
Representative case of HHT with developed A-V shunts: (**a**,**b**) Color Doppler US reveals many dilatations of HA (arrows) and vascular shunts within the liver. (**c**) The presence of a large A-V shunt (arrows) is confirmed using CEUS.

Here, to understand these vascular shunts, we briefly discuss HHT. It is a multiorgan genetic angiodysplastic disease characterized by visceral vascular malformations and is diagnosed based on the Curacao criteria [46]. It affects not only the brain, lungs, gastrointestinal tract, and nasal mucosa, but also the liver [45,47]. It is well known that HHT produces many kinds of intrahepatic vascular shunts (Figure 8). Among these shunts, the A-V shunt is clinically the most important. The marked development of the A-V shunt results in ischemia and necrosis of the bile duct and portal vein wall due to stealing of arterial blood flow that is purposed to nourish the bile duct and portal walls. This leads to cholangitis, biloma, and liver abscess formation (Figure 9) [45,48]. More importantly, the increased blood flow draining into the cardiac system results in cardiac overload, which in turn induces chronic heart failure [45,49]. Of note is the fact that the development of each shunt is not even; therefore, the development of the A-V shunt also varies greatly among HHT individuals. From the diagnostic point of view, although color Doppler US can provide a rough image of intrahepatic vascular shunts, it is necessary to confirm whether the vessels in question are merely in close proximity to each other or whether a real shunt exists. CEUS facilitates this differentiation and the diagnosis of A-V shunts.

### 4.2. Congenital P-V Shunts

**Figure 10:**
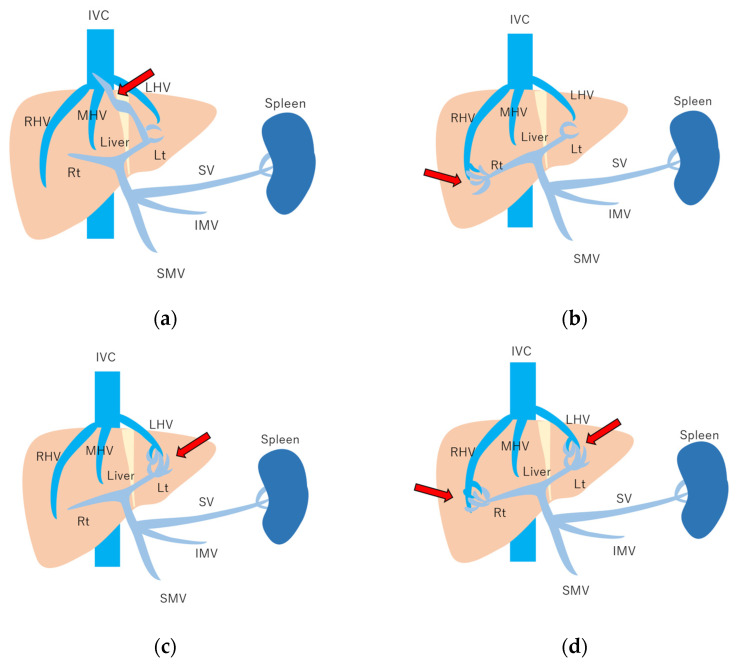
Schematic drawing of 4 types of congenital P-V shunt: (**a**) Type 1: Single large channel communicating with the right portal vein. (**b**) Type 2: Multiple communications between peripheral portal branches and a hepatic vein in one segment. (**c**) Type 3: Connection of hepatic veins and a portal vein through an aneurysm. (**d**) Type 4: Multiple communications between peripheral portal branches and peripheral hepatic branches. Arrows, abnormal communication site; IVC, inferior vena cava; LHV, left hepatic vein; MHV, middle hepatic vein; RHV, right hepatic vein; Lt, left hepatic lobe; Rt, right hepatic lobe; SV, splenic vein; IMV, inferior mesenteric vein; SMV, superior mesenteric vein. (Cited from [51] with adequate modification.)

**Figure 11:**
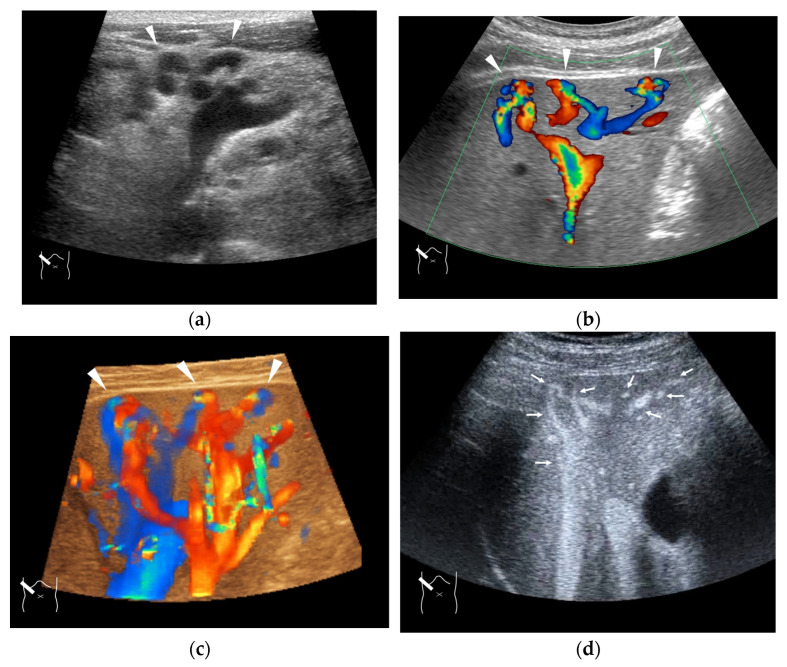
Representative case of congenital P-V shunt (type 2) in segment 6: (**a**) B-mode US. (**b**) Color Doppler US. (**c**) Three-dimensional color Doppler US reveals P-V shunts (arrow heads). (**d**) CEUS. It can be seen that all peripheral HV branches are simultaneously enhanced (arrows).

In the clinical setting, it is not so unusual to encounter intrahepatic small-sized P-V shunts in asymptomatic cases. In contrast, the occurrence of large intrahepatic P-V shunts is relatively rare and may induce hepatic encephalopathy [50], and most of the symptomatic P-V shunts are usually congenital, although clinical manifestations occur in older age groups. Currently, there are four main types of congenital P-V shunts (Figure 10) [51]. Of these, the most common type is the right lobe type, which is characterized by a cluster of multiple fine shunts communicating with portal veins of small diameter and HVs in the posterior segment of the right hepatic lobe, as shown in Figure 11. The role of CEUS in the diagnosis of congenital P-V shunts is to demonstrate the direct outflow from portal veins to HVs, regardless of their size or tortuousness.

### 4.3. Budd–Chiari Syndrome

**Figure 12:**
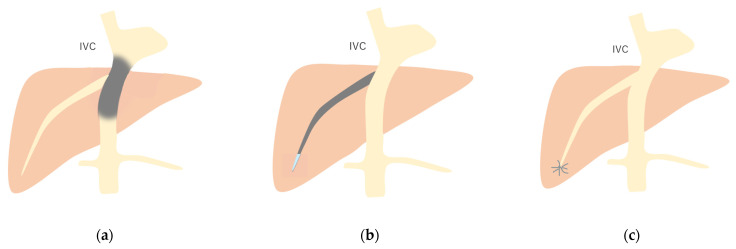
Schematic drawing of 3 types of Budd–Chiari syndrome: (**a**) Type 1: Budd–Chiari syndrome due to membranous obstruction of IVC. (**b**) Type 2: This is due to the obstruction of a large intrahepatic vein. (**c**) Type 3: This is due to the obstruction of localized small HV branches. Gray area, stenosis or obstruction site. (Cited from [52] with adequate modification.)

**Figure 13:**
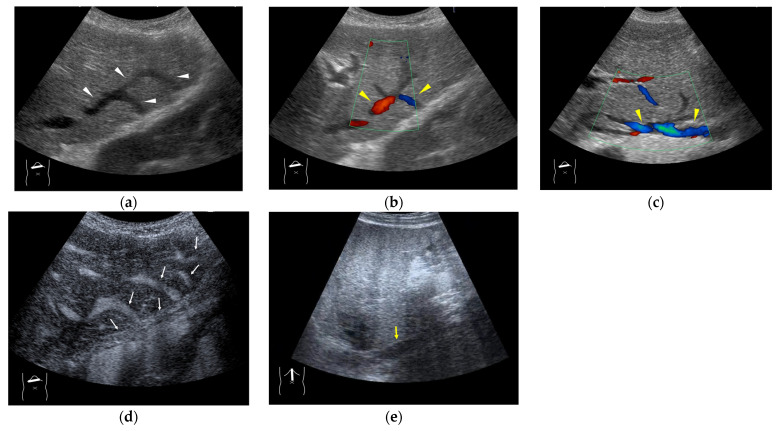
Representative case of Budd–Chiari syndrome (type 1): (**a**) B-mode US shows many venous collaterals (arrow heads) in the left lobe. (**b**,**c**) Color Doppler US shows slow flows in these collaterals (yellow arrow heads). (**d**) CEUS confirms communication between these collaterals and intracapsular collaterals (arrows). (**e**) It confirms no blood flow in obstructed IVC (yellow arrow).

In a small number of cases, the cause of intrahepatic V-V shunts is congenital; they are seen mainly in a small number of HHT diseases. However, most of the V-V shunt cases are almost always acquired due to some form of HV occlusion, which can be clinically divided into two main types: HV thrombus and compression of a mass lesion (Figure 12) [52,53]. The V-V shunt is formed when an accidental stenosis or obstruction occurs at any point between the intrahepatic HV and inferior vena cava. On the other hand, in patients with a hepatic mass lesion, HVs are easily compressed by the mass because they have a thinner wall than other hepatic vessels (arteries and portal veins) and are easily affected by external pressure [54]. Simply speaking, V-V shunts occur when the responsible mass lesion, whether benign or malignant, exerts a large external compression force. Compared with the other intrahepatic shunts, V-V shunts have no important clinical implications. Thus, in the clinical setting, the precise diagnosis of V-V shunts serves to exclude the possibility of other shunts. The role of CEUS is to confirm the stenosis or complete occlusion of HVs and IVC; the result affects subsequent treatment strategies (Figure 13).

### 4.4. Hemodynamic Abnormalities in Liver Abscess

Liver abscesses are often difficult to diagnose and treat because of the variety of causative organisms, clinical symptoms, and imaging findings [55,56]. One of the most important clinical considerations is the early detection of severe cases [56,57]. The imaging findings of severe abscesses include vascular involvement as well as gas production and hypervascularity due to severe inflammation [58]. Portal vein thrombus is considered a finding that may lead to further development of the disease [59], but HV involvement is of even greater diagnostic significance. It has frequently been reported that septic shock occurs in liver abscess patients with severe clinical conditions [60,61]. It is not irrational to think that severe hepatic infection disseminates through hepatic venous blood flow to the systemic circulation; thus, the visualization of the hepatic venous flow draining from the liver abscess lesion is clinically important. When using CEUS, the diagnosis is easy to make because the contrast medium that borders the abscess lesion itself and then immediately drains into the HV.

### 4.5. Hemodynamic Abnormalities in Liver Necrosis

**Figure 14:**
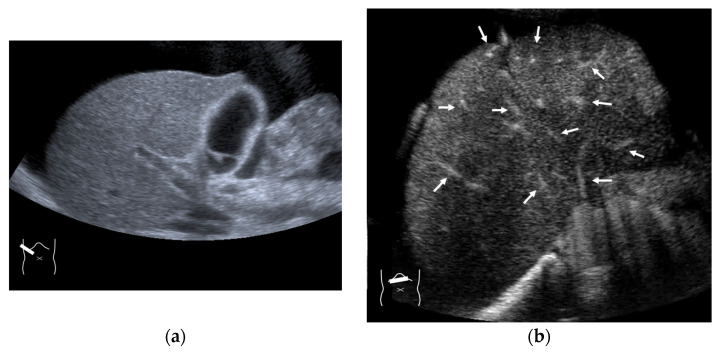
Representative case of chemotherapy-induced sinusoidal obstruction syndrome: (**a**) B-mode US shows that the liver has a homogeneous texture. (**b**) CEUS shows that all hepatic veins and portal veins are simultaneously enhanced (arrows).

The main cause of acute hepatic necrosis is severe acute hepatitis [62,63]. The combination of clinical manifestations, biochemical data, and diagnostic imaging should be synthesized to assess the rapidly changing clinical condition of the patient without time delay. In many cases, it is difficult to provide a detailed picture of the patient’s condition using imaging, especially when using B-mode US only, which is frequently utilized to assess the patient’s physical condition: it focuses mainly on the liver size, the irregular hepatic surface, and the presence of ascites [63]. However, except in very advanced cases, the parenchymal pattern is almost uniform, and the blood flow appears to be macroscopically normal. Although there have been few reports, CEUS is expected to be a solution to this diagnostic dilemma. In acute liver failure, some US features have been reported, including increased HA velocity in Doppler US and increased parenchymal shear wave values in US elastography [64]. An earlier hepatic parenchymal enhancement in CEUS has also been reported [64]. Kuroda speculated that hepatic microcirculation disturbances and destruction of the sinusoidal structure due to massive necrosis may decrease the low-pressure portal venous blood flow, which is immediately compensated by the HA blood flow increase, through the formation of intrahepatic shunts. These vascular changes are thought to induce a very fast HV arrival time. This reduced HV transit time can be clearly detected by using CEUS. Although this reduced HV transit time is highly diagnostic, it is not very specific to liver necrosis. It is also seen in sinusoid obstruction syndrome (SOS) (Figure 14) [65,66,67] or liver cirrhosis [20,68]. Thus, the most important way to make use of this useful information is to interpret it in conjunction with the clinical data.

### 4.6. Hemodynamic Abnormalities in Acute Cholecystitis

**Figure 15:**
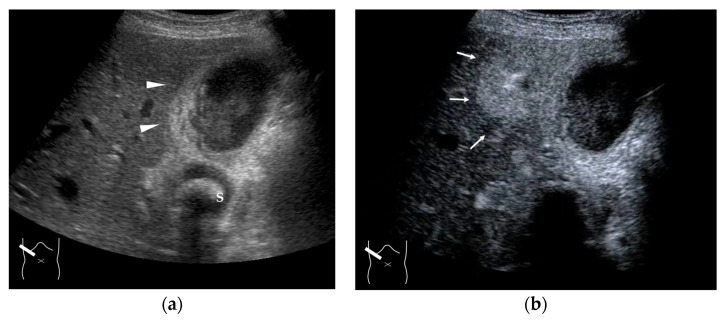
Representative case of acute cholecystitis: (**a**) B-mode US shows the gallbladder wall to be thickened (arrow heads). (**b**) CEUS shows a triangular or oval hyper-enhanced zone (arrows) near the gallbladder in the arterial phase. S, gallstone.

The gallbladder is fed by the cystic artery, which is a branch of the hepatic artery, but the draining vessels are extremely fine (Figure 15). When arterial flow to the gallbladder wall is increased in acute cholecystitis, these draining fine vessels become dilated; this is frequently imaged on CT findings in acute cholecystitis cases as a hypervascular zone surrounding the gallbladder wall [69,70,71]. In acute cholecystitis, this finding can also be seen in the CEUS image. In the arterial phase, CEUS shows that microbubbles quickly enhance the gallbladder wall and then drain the liver parenchyma in segment S4 or S5 in a thread-like pattern [70]. Most of the images are triangular or oval in shape with the gallbladder as the base (Figure 15), and unlike CT, this triangular liver parenchymal enhancement time is extremely short. This may be due to the characteristics of the CEUS microbubble, which moves through the microvasculature in a short period of time, rather than moving into the interstitium and remaining there for a relatively long time as in CT. However, this CEUS finding can be used to determine the severity of cholecystitis, which is an aspect that deserves attention.

## 5. Future Perspectives

As described in this review, the observation of HVs using CEUS can greatly contribute to the understanding of the pathophysiology of liver diseases and can improve the accuracy of diagnostic assessments. However, an understanding of the mechanism of each finding is essential for widening the use of CEUS for this purpose. This is not necessarily appropriate for general clinical practice, as CEUS diagnosis requires a fairly high level of expertise. Therefore, in the future, the widespread use of the automatic analysis of CEUS findings using AI [16] will be necessary to some extent for the general use of the CEUS diagnosis. To this end, further simplification and faster application of the time–intensity curve and a wide range of parametric image analyses using the results of the time–intensity curve are inevitable [72,73]. In addition, with current US equipment, it is not always easy to observe the hepatic veins in the same cross-section as other vessels (arteries and portal veins). The restricted field of view, which is always a drawback of US diagnosis in general, applies to CEUS diagnosis as well and makes it more difficult. The development of wide scanning and the 3D display [74,75] compensates for this disadvantage to some extent.

## 6. Conclusions

In this study, the current status of HV diagnosis using CEUS is described primarily through the use of a literature review. In order to maintain the CEUS diagnostic accuracy, it is essential to fully understand the pathomechanism of each CEUS finding. The basic concepts of CEUS and transit time are especially important. Additionally, it is also necessary to expand our knowledge on pathology, such as that involving tumor vessels and drainage vessels.
